# Strategies to Lower In-Hospital Mortality in STEMI Patients with Primary PCI: Analysing Two Years Data from a High-Volume Interventional Centre

**DOI:** 10.1155/2019/3402081

**Published:** 2019-10-01

**Authors:** Alexandru Burlacu, Grigore Tinica, Igor Nedelciuc, Paul Simion, Bogdan Artene, Adrian Covic

**Affiliations:** ^1^Head of Department of Interventional Cardiology—Cardiovascular Diseases Institute, “Grigore T. Popa” University of Medicine, Iasi, Romania; ^2^Department of Cardiovascular Surgery—Cardiovascular Diseases Institute, “Grigore T. Popa” University of Medicine, Iasi, Romania; ^3^Department of Interventional Cardiology—Cardiovascular Diseases Institute, Iasi, Romania; ^4^Nephrology Clinic, Dialysis and Renal Transplant Center—“C.I. Parhon” University Hospital, “Grigore T. Popa” University of Medicine, Iasi, Romania; ^5^The Academy of Romanian Scientists (AOSR), Iasi, Romania

## Abstract

**Objectives:**

We aimed to analyse data from our high-volume interventional centre (>1000 primary percutaneous coronary interventions (PCI) per year) searching for predictors of in-hospital mortality in acute myocardial infarction (MI) patients. Moreover, we looked for realistic strategies and interventions for lowering in-hospital mortality under the “*5 percent threshold.” Background*. Although interventional and medical treatment options are constantly expanding, recent studies reported a residual in-hospital mortality ranging between 5 and 10 percent after primary PCI. Current data sustain that mortality after ST-elevation MI will soon reach a point when cannot be reduced any further.

**Methods:**

In this retrospective observational single-centre cohort study, we investigated two-year data from a primary PCI registry including 2035 consecutive patients. Uni- and multivariate analysis were performed to identify independent predictors for in-hospital mortality.

**Results:**

All variables correlated with mortality in univariate analysis were introduced in a stepwise multivariate linear regression model. Female gender, hypertension, depressed left ventricular ejection fraction, history of MI, multivessel disease, culprit left main stenosis, and cardiogenic shock proved to be independent predictors of in-hospital mortality. The model was validated for sensitivity and specificity using receiver operating characteristic curve. For our model, variables can predict in-hospital mortality with a specificity of 96.60% and a sensitivity of 84.68% (*p* < 0.0001, AUC = 0.93, 95% CI 0.922–0.944).

**Conclusions:**

Our analysis identified a predictive model for in-hospital mortality. The majority of deaths were due to cardiogenic shock. We suggested that in order to lower mortality under 5 percent, focus should be on creating a cardiogenic shock system based on the US experience. A shock hub-centre, together with specific transfer algorithms, mobile interventional teams, ventricular assist devices, and surgical hybrid procedures seem to be the next step toward a better management of ST-elevation MI patients and subsequently lower death rates.

## 1. Introduction

Myocardial infarction (MI) remains one of the leading causes of global cardiovascular burden [1]. Even if numerous efforts have been made to increase awareness, prevention, and management of acute MI, it still has a high incidence—ST-elevation myocardial infarction (STEMI) accounting for high mortality and morbidity rates [2]. Since 2008, when “*Stent for Life*” initiative expanded throughout all Europe (including Eastern countries), death rates in STEMI decreased to a plateau [3].

Although interventional and medical treatment options are constantly expanding, recent studies reported a residual in-hospital mortality ranging between 5 and 10 percent [4–6]. Furthermore, an expert opinion from the European Society Cardiology 2017 Congress stated that “*we may soon reach a point when mortality after STEMI cannot be reduced any further*” [7]. According to recent data presented from SWEDEHEART (Sweden's online cardiac registry [8]), there has been little change in mortality in the past 10 years in Sweden, which implies that “*it will now become very difficult to further decrease mortality*” [9]. Optimization of diagnostic and interventional treatment delays, as well as innovating novel drugs and treatment concepts, a better medical education and primary prevention of atherosclerotic disease are currently the envisioned solutions for lowering mortality.

In this paper, we aimed to analyse two years data from a high volume single interventional centre serving for the eastern part of Romania (the only primary percutaneous coronary interventions (PCI) facility for at least 7 million inhabitants, with more than 1000 primary PCI per year) searching for predictors of in-hospital mortality. Also, we intended to identify and summarize realistic strategies and interventions to lower mortality under the “*5 percent threshold*” in STEMI.

## 2. Materials and Methods

### 2.1. Study Design and Patient Population

This is a retrospective observational single-centre cohort study. Institutional ongoing registry of primary PCI procedures was reviewed from 01 January 2017 to 01 January 2019, and 2035 consecutive patients were selected. Our facility is a high-volume tertiary centre focused on coronary interventions (∼5000 procedures/year) accounting for 8 districts in the north-eastern part of Romania, which provides a 24-hour primary PCI service to a population of 7,000,000 inhabitants. Our registry is affiliated to RO-STEMI (Romanian Registry for ST-segment Elevation Myocardial Infarction) [10].

The study's protocol was approved by the ethical committee (review board) of Cardiovascular Diseases Institute “George I.M. Georgescu” Iasi. The analysis was conducted according to Declaration of Helsinki. No sex-based or racial/ethnic-based differences were present.

### 2.2. Definitions and Data Collection

ST-Elevation Myocardial Infarction (STEMI) was defined using the ESC criteria (clinical evidence of acute myocardial ischemia and with detection of a rise and/or fall of cTn values, coexisting with symptoms, new ECG changes, and imaging or angiographic evidence attributable to an ischemic etiology), according to the Fourth Universal Definition of Myocardial Infarction [11].

Cardiogenic shock was defined as systolic blood pressure <90 mmHg >30 min or vasopressors required to achieve ≥90 mmHg secondary to severe ventricular dysfunction associated with signs of impaired organ perfusion (e.g., altered mental status, cold skin and extremities, oliguria, or serum lactate >2.0 mmol/L) [12].

All patients were routinely treated with double antiplatelet therapy (dosage regimen according to ESC MI guidelines) and with an intravenous bolus of unfractionated heparin (100 U/kg body weight). IV administration of eptifibatide was left to the discretion of the operator. *β*-Adrenergic blockers, ACE inhibitors, and statins were used as standard therapy, if not contraindicated.

Procedural characteristics were assessed by the interventional cardiologist at the time of the PCI, and coronary lesions were evaluated according to the ACC/AHA classification [13]. Severity indicators of myocardial infarction (e.g., multivessel disease, culprit left main (LM), culprit proximal anterior descending artery, myocardial rupture, and in-stent thrombosis) were also included. More than 50% stenosis of left main artery (LM), and more than 75% stenosis of left anterior descending artery (LAD), left circumflex artery (LCX), right coronary artery (RCA), and main branch of these vessels in addition to ischemic symptoms or ischemic evidence, was considered indication for percutaneous coronary intervention.

Clinical data were obtained from patient's medical charts. Recorded risk factors included age (>70-year-old), sex, diabetes, dyslipidemia, smoking history, hypertension, chronic kidney disease (eGFR <60 mL/min/m^2^), depressed myocardial ejection fraction (<35%), previous MI, and prehospital cardiac resuscitation.

All death causes were examined by two of the investigators.

### 2.3. Statistical Analysis

Categorical variables were compared between the two groups using the *χ*2 tests. Continuous data were reported as means and standard deviations and were compared using the Student's *t*-tests. Potential predictive factors for in-hospital mortality were identified using univariate analysis. Significant variables were included in a stepwise multivariate model to determine independent predictors of in-hospital mortality, presented as OR and 95% CI, with a *p*-value <0.05 considered as significant. C statistics and receiver operating curve (ROC) were used for the model, including predictors in order to evaluate the predictive performance of both scores for in-hospital mortality. Youden index values were computed.

All statistical analysis was performed using SPSS 20.0 (SPSS Inc, Chicago, IL, USA).

## 3. Results

All recorded data from the 2035 patients were analysed. The mean age was 60 ± 10.2 years; 56.3% (*n* = 1145) of the enrolled patients were older than 70 years, and 38.9% (*n* = 792) were female.

67% of pPCI patients (*n* = 1366) were smokers (or had a history of smoking over 10 pack-years), 27.4% (*n* = 558) were diabetics, 56.3% (*n* = 1146) had dyslipidemia, 57.3% (*n* = 1167) had hypertension, 3% had a history of previous MI (*n* = 61), and 19.8% (*n* = 394) were known with chronic kidney disease (CKD).

At admission, 19.8% (*n* = 403) had left ventricular ejection fraction <35%, and 10% (*n* = 204) presented with cardiogenic shock. Following coronarography, multivessel disease (defined by stenosis >50% in two or more epicardial coronary arteries) was identified in 21.1% (*n* = 430), culprit proximal LAD in 20.2% (*n* = 412), and culprit LM in 11.2% (*n* = 226) of patients. Myocardial rupture was recorded in 0.6% (*n* = 12) of cases and acute in-stent thrombosis in 0.4% (*n* = 10) both with 100% in-hospital mortality rate due to refractory cardiogenic shock. 2 patients died from acute aortic dissection with diffuse extension in the left coronary artery. Descriptive statistics is shown in Table 1.

Overall in-hospital mortality was 6.1% (*n* = 124), and it was most frequent in patients presenting with cardiogenic shock (*p* < 0.001, OR 37.81, 95% CI 20–60). In cardiogenic shock patients, the mortality rate was as high as 48%, which means that one out of two patients with cardiogenic shock died. Almost all patients with myocardial rupture and in-stent thrombosis died.

Following univariate analysis, total in-hospital mortality was also associated with advanced age (>70 years), female gender, smoking, diabetes mellitus, elevated blood pressure, dyslipidemia, reduced left ventricular ejection fraction <35%, prehospital cardiac resuscitation, history of MI, multivessel disease, culprit proximal LAD, and culprit LM (Table 2).

All variables correlated with mortality in univariate analysis were introduced in a separate stepwise multivariate linear regression model. Multivariate analysis of significant variables revealed that after adjusting for all clinical variables, female gender, hypertension, depressed left ventricular ejection fraction, history of MI, multivessel disease, culprit LM, and cardiogenic shock remained independent predictors of in-hospital mortality (Table 3).

**Figure 1:**
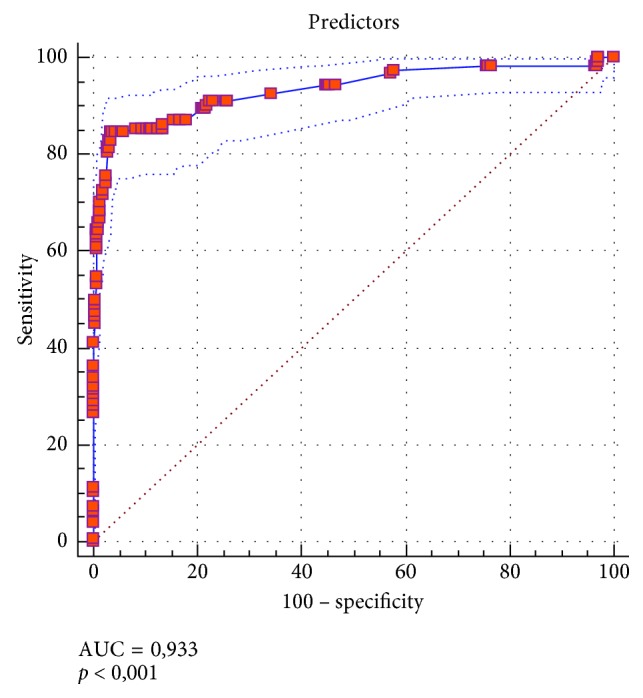
Performance of the model for predicting in-hospital mortality.

The model was also validated for sensitivity and specificity using receiver operating characteristic (ROC) curve. For our model, the significant independent variables can predict in-hospital mortality with a specificity of 96.60% and a sensitivity of 84.68% (*p* < 0.0001, AUC = 0.93, 95% CI 0.922–0.944) (Figure 1, Table 4).

## 4. Discussions

The present study evaluated data from more than 2000 primary PCI consecutive patients instrumented in our centre in the last 2 years. We reported that besides gender (female sex), previous myocardial infarction, a cardiovascular risk factor (high blood pressure), and four variables depicting extensive and severe MI (LM disease, multivessel disease, cardiogenic shock, and LVEF <35%) proved to be independent predictors for in-hospital mortality in STEMI.

The above results are concordant with previous smaller researches showing almost similar predictors [4–6, 10, 14].

Our endeavour not only intended to elaborate a predictive model for mortality but also to identify ways to reduce in-hospital mortality. To date, there is no algorithm or specific guideline tackling these problems, nor a recommended auditing protocol to assess the number and causes of deaths (in each primary PCI facility). What is a “*tolerable”* percentage of mortality and from which threshold one can trigger an alarm questioning the quality of pPCI network/healthcare management? We suggest that the next ESC STEMI guideline should include recommendations for reporting, evaluating, and auditing the numbers and etiologies of death in every primary PCI centre.

A recent study on STEMI patients with cardiogenic shock [15] revealed that the time elapsing from the first medical contact to primary PCI is a strong predictor of an adverse outcome in this group of patients. On the other hand, the same trial underlined that haemodynamic instability resulted in treatment delay [15]. Thus, to lower mortality in a STEMI patient with cardiogenic shock, multivessel disease, and with LVEF lower than 35% seems like a paradox and a (still) unsolved puzzle: on the one hand, one needs a shorter time to balloon; on the other hand, this scenario generates a longer time due to necessity of more medical manoeuvers or stabilisation intervals.

A few questions are emerging from this situation: (a) How can we prevent a patient to develop cardiogenic shock? (b) How can we improve survival in a patient with cardiogenic shock and primary PCI?

**Figure 2:**
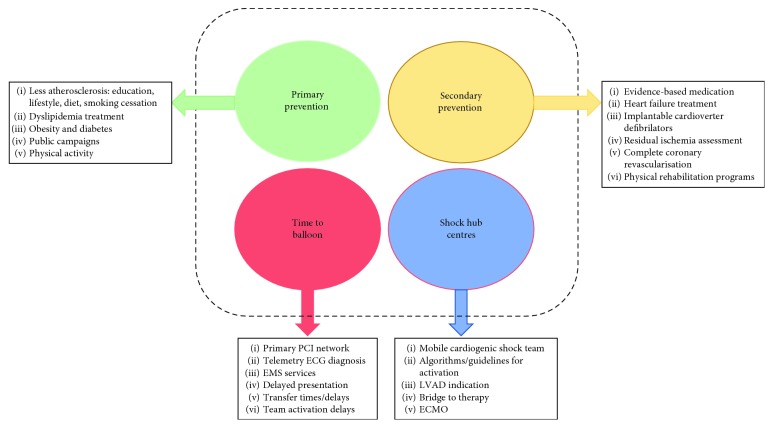
Strategies to lower in-hospital mortality in primary PCI patients.

A well-known fact contributing to cardiogenic shock survival is the time from the first symptoms to balloon. Besides patients' medical education (recognizing the pain and early presentation to a medical examination), the benefit of early ECG on short-term in-hospital survival most probably accounts for faster decision processes during patient management. These elements seem to be key determinants for survival in STEMI patients with cardiogenic shock [15] (Figure 2).

Recently, significant progresses were done in the USA for the formation of regionalized systems of care for specific cardiovascular emergencies, especially cardiogenic shock. The idea is to make a step forward beyond a common primary PCI system: “*lifeline-supported pathway for the development of integrated regionalized cardiogenic shock systems of care*” [16]. This concept of regional systems for treating cardiogenic shock patients includes a hub centre different than tertiary primary PCI facilities. Currently, this project is not worldwide implemented and not (yet) endorsed by the guidelines.

The existing facilities underlined the need for an early dialogue (within 12 hours of shock) between the referring and accepting centres to determine the viability of the patients for advanced therapies and the suitability for transfer and developed a management algorithm. Implementation of this network was associated with a 66% survival rate, higher than the 25% historical survival rate [16–19]. The transfer between primary PCI institutions and regional referral shock centres should be organized and monitorized by the “*mobile cardiogenic shock teams*” [20]. The traveling mechanical support team concept uses mobile ECMO devices as a bridge to more advanced therapies as bridge-to-transplantation or recovery [21].

This paradigm of “*fourth level centres*” developed having more advanced therapies and resources for cardiogenic shock (as cardiac surgery, percutaneous ventricular assist devices, implantable VAD, ECMO, and ECMO-mobile teams), which usually are not available in contemporary 24 h/7 d/primary PCI facilities [16].

We are aware that without these special resources our centre cannot lower in-hospital mortality in primary PCI STEMI patients under an “*accepted reasonable*” threshold of 5%. New algorithms suggest that patients presenting to smaller spoke centres without PCI capabilities should be immediately transferred to the nearest PCI facility, or a shock mobile unit should be requested from the hub CS centre, depending on the patient's clinical status and anticipated travel time [16]. Focusing only on primary PCI issues (as complete *versus* only culprit vessel revascularisation [22], TIMI slow/no-flow management [23]) seems not to solve the still high in-hospital mortality.

Many of the most successful STEMI systems actively include advanced cardiogenic shock protocols [24]. Presently, the European Guidelines focus mostly on cardiogenic shock treatment, but there is an increasing need for a new guideline with protocols for a shock management system (centres, resources, mobile teams, advanced therapies, coordinated approach, and auditing) as the US already developed [24].

We are aware of the limitations of our proposed model, as all previously published models reported limitations: “*lack of a CS-specific derivation population, external validation, dynamic application (i.e., single point in time only), applicability to all CS types, and capture of all potentially prognostic clinical, laboratory, hemodynamic, imaging, and biomarker data*” [16]. The investigators of the CardShock study developed a risk prediction score for short-term mortality in cardiogenic shock due to all etiologies (not only the ischemic cause). Four out of seven predictors were similar to those reported by our team, the other three being confusion, blood lactate levels, and prior coronary artery bypass [25].

We realize that we did not include all possible variables in multivariate analysis (for example, we excluded all time-related variables, as there are very good recent reports on this topic [15]). However, our solid and reliable data reflect the very limitations of a primary PCI system dealing with complex cardiogenic shock cases bearing high mortality.

## 5. Conclusions

Our analysis derived from a single centre primary PCI experience identified a predictive model for in-hospital mortality. Besides hypertension and female sex, the other variables were related to cardiogenic shock. In fact, the majority of the in-hospital deaths recorded in our centre had cardiogenic shock. We suggested that in order to lower mortality under the 5 percent threshold, our focus should be on creating a cardiogenic-shock system based on the US experience. A shock hub centre, together with specific transfer algorithms, mobile interventional teams, ventricular assist devices, and surgical hybrid procedures, seems to be the next step toward a better management of STEMI patients and subsequently lower death rates.

## Figures and Tables

**Table 1:** Baseline and clinical characteristics of the patients included in the analysis.

Characteristic	Total		In-hospital deaths		Percentage of death inside each category
	*n* = 2035, 100%		*n* = 124, 6.1%		
Age >70 y, *n* (%)	1145	56.3%	78	3.8%	6.9
Female gender, *n* (%)	792	38.9%	84	4.2%	10.6
Smoking, *n* (%)	1366	67.1%	104	5.1%	7.6
Diabetes mellitus	558	27.4%	72	3.5%	12.9
Hypertension	1167	57.3%	110	5.4%	9.4
Dyslipidemia	1146	56.3%	120	5.9%	10.2
Chronic kidney disease	394	19.4%	18	0.9%	4.6
LVEF <35%	403	19.8%	112	5.5%	26.2
Primary resuscitation	150	7.4%	32	1.6%	21.3
Previous MI	61	3%	25	1.23%	41
Multivessel disease	430	21.1%	85	4.2%	19.8
Proximal LAD	412	20.2%	53	2.6%	12.9
LM	227	11.2%	34	1.7%	15
Cardiogenic shock	204	10%	109	5.35%	48.9
Myocardial rupture	12	0.6%	12	0.6%	100
In-stent thrombosis	10	0.4%	10	0.5%	100
Hospitalisation days	5.17 ± 1.94		1.94 ± 0.239		

**Table 2:** Univariate analysis of selected predictors for in-hospital mortality in STEMI patients.

Characteristic	In-hospital mortality	OR	95% CI	*p*
Age >70 y, *n* (%)	**78 (6.9%)**	**1.68**	**1.1–2.4**	**0.08**
Female gender, *n* (%)	**84 (10.6%)**	**0.28**	**0.19–0.41**	**<0.001**
Smoking, *n* (%)	**104 (7, 6%)**	**2.67**	**1.64–4.35**	**<0.001**
Diabetes, *n* (%)	**72 (12.9%)**	**4.06**	**2.80–5.88**	**<0.001**
Hypertension, *n* (%)	**110 (9.4%)**	**6.34**	**3.61–11.15**	**<0.001**
Dyslipidemia, *n* (%)	**120 (10.2%)**	**2.68**	**1.75–4.10**	**<0.001**
CKD, *n* (%)	18 (4.6%)	0.69	0.41–1.15	NS
LVEF <35%, *n* (%)	**112 (26.2%)**	**11.86**	**7.93–17.766**	**<0.001**
Primary resuscitation	**32 (21.3%)**	**5.28**	**3.39–8.23**	**<0.001**
Previous MI	**25 (41%)**	**13.15**	**7.59–22.77**	**<0.001**
Multivessel disease	**85 (19.8%)**	**9.89**	**6.65–14.70**	**<0.001**
Proximal LAD	**53 (12.9%)**	**3.22**	**2.21–4.69**	**<0.001**
LM	**34 (15%)**	**3.36**	**2.20–5.12**	**<0.001**
Cardiogenic shock	**109 (48.9%)**	**41.72**	**26.93–64.63**	**<0.001**
Hospitalisation days	5.17 ± 1.94	1.08	0.98–1.20	NS

**Table 3:** Independent predictors for in-hospital mortality in STEMI patients by multivariate analysis.

Characteristic	OR	95% CI	*p*
Age >70 y	0.59	0.28–1.21	NS
Female gender	**2.92**	**1.58–5.38**	**0.001**
Smoking	0.86	0.39–1.89	NS
Diabetes	0.61	0.30–1.25	NS
Hypertension	**15.77**	**5.0–40.43**	**<0.001**
Dyslipidemia	0.29	0.11–0.78	0.078
LVEF <35%	**16.00**	**7.73–30.09**	**<0.001**
Primary resuscitation	0.65	0.30–1.38	NS
Previous MI	**5.23**	**2.20–12.42**	**<0.001**
Multivessel disease	**4.68**	**2.45–8.94**	**<0.001**
Proximal LAD	0.72	0.39–1.32	NS
Culprit LM	**1.81**	**1.12–2.90**	**0.014**
Cardiogenic shock	**37.81**	**20.5–60.52**	**<0.001**

**Table 4:** Area under the ROC curve and Youden index values for our model.

Area under the ROC curve (AUC)	0.933
Standard error	0.0156
95% confidence interval	0.922 to 0.944
*Z* statistic	27.738
Significance level *p* (area = 0.5)	<0.0001
Youden index *J*	0.8128
Associated criterion	≤0.868103239
Sensitivity	84.68
Specificity	96.60

## Data Availability

The data used to support the findings of this study are available from the corresponding author upon request.

## References

[1] Moran A. E., Forouzanfar M. H., Roth G. A. (2014). The global burden of ischemic heart disease in 1990 and 2010: the global burden of disease 2010 study. *Circulation*.

[2] Yeh R. W., Sidney S., Chandra M., Sorel M., Selby J. V., Go A. S. (2010). Population trends in the incidence and outcomes of acute myocardial infarction. *New England Journal of Medicine*.

[3] Kaifoszova Z., Kala P., Alexander T. (2014). Stent for life initiative: leading example in building STEMI systems of care in emerging countries. *EuroIntervention*.

[4] Ali M., Lange S. A., Wittlinger T., Lehnert G., Rigopoulos A. G., Noutsias M. (2018). In-hospital mortality after acute STEMI in patients undergoing primary PCI. *Herz*.

[5] Kytö V., Sipilä J., Rautava P. (2015). Gender and in-hospital mortality of ST-segment elevation myocardial infarction (from a multihospital nationwide registry study of 31,689 patients). *The American Journal of Cardiology*.

[6] García-García C., Ribas N., Recasens L. L. (2017). In-hospital prognosis and long-term mortality of STEMI in a reperfusion network. “Head to head” analisys: invasive reperfusion vs optimal medical therapy. *BMC Cardiovascular Disorders*.

[7] Danchin N. (2017). *ESC 2017: Further Decreasing Mortality after STEMI Will be Difficult*.

[8] https://www.ucr.uu.se/swedeheart/

[9] Jernberg T., Wallentin L., Alfredsson J. SWEDEHEART—no changes in survival after acute myocardial infarction in the last decade-new data.

[10] Cretu D. E., Udroiu C. A., Stoicescu C. I., Tatu-Chitoiu G., Vinereanu D. (2015). Predictors of in-hospital mortality of ST-segment elevation myocardial infarction patients undergoing interventional treatment. An analysis of data from the RO-STEMI registry. *Maedica (Buchar)*.

[11] Thygesen K. (2019). “Ten commandments” for the fourth Universal definition of myocardial infarction 2018. *European Heart Journal*.

[12] Thiele H., Ohman E. M., Desch S., Eitel I., de Waha S. (2015). Management of cardiogenic shock. *European Heart Journal*.

[13] Ryan T. J., Faxon D. P., Gunnar R. M. (1988). Guidelines for percutaneous transluminal coronary angioplasty. A report of the American college of cardiology/American heart association task force on assessment of diagnostic and therapeutic cardiovascular procedures (subcommittee on percutaneous transluminal coronary angioplasty). *Circulation*.

[14] Gale C. P., Manda S. O. M., Batin P. D., Weston C. F., Birkhead J. S., Hall A. S. (2008). Predictors of in-hospital mortality for patients admitted with ST-elevation myocardial infarction: a real-world study using the myocardial infarction National Audit Project (MINAP) database. *Heart*.

[15] Scholz K. H., Maier S. K. G., Maier L. S. (2018). Impact of treatment delay on mortality in ST-segment elevation myocardial infarction (STEMI) patients presenting with and without haemodynamic instability: results from the German prospective, multicentre FITT-STEMI trial. *European Heart Journal*.

[16] van Diepen S., Katz J. N., Albert N. M. (2017). Contemporary management of cardiogenic shock: a scientific statement from the American heart association. *Circulation*.

[17] Helman D. N., Morales D. L. S., Edwards N. M. (1999). Left ventricular assist device bridge-to-transplant network improves survival after failed cardiotomy. *The Annals of Thoracic Surgery*.

[18] Hasin Y., Danchin N., Filippatos G. S. (2005). Recommendations for the structure, organization, and operation of intensive cardiac care units. *European Heart Journal*.

[19] Casella G., Zagnoni S., Fradella G. (2017). The difficult evolution of intensive cardiac care units: an overview of the BLITZ-3 registry and other Italian surveys. *BioMed Research International*.

[20] Beurtheret S., Mordant P., Paoletti X. (2013). Emergency circulatory support in refractory cardiogenic shock patients in remote institutions: a pilot study (the cardiac-RESCUE program). *European Heart Journal*.

[21] Jaroszewski D. E., Kleisli T., Staley L. (2011). A traveling team concept to expedite the transfer and management of unstable patients in cardiopulmonary shock. *The Journal of Heart and Lung Transplantation*.

[22] Hochman J. S., Sleeper L. A., Webb J. G. (1999). Early revascularization in acute myocardial infarction complicated by cardiogenic shock. SHOCK investigators. Should we emergently revascularize occluded coronaries for cardiogenic shock. *New England Journal of Medicine*.

[23] Windecker S., Kolh P., Alfonso F. (2014). ESC/EACTS guidelines on myocardial revascularization: the task force on myocardial revascularization of the european society of Cardiology (ESC) and the European association for cardio-thoracic surgery (EACTS)developed with the special contribution of the european association of percutaneous cardiovascular interventions (EAPCI). *European Heart Journal*.

[24] Graham K. J., Strauss C. E., Boland L. L. (2012). Has the time come for a national cardiovascular emergency care system?. *Circulation*.

[25] Harjola V.-P., Lassus J., Sionis A. (2015). Clinical picture and risk prediction of short-term mortality in cardiogenic shock. *European Journal of Heart Failure*.

